# Supplementary material for: Unveiling Health Inequalities: Exploring Metabolic Dysfunction in Rural Roma Communities
Source: Healthcare (Basel). 2024 Apr 11;12(8):816. doi: 10.3390/healthcare12080816 (PMC11050184; doi:10.3390/healthcare12080816)
Supplement: Supplementary file 1 [file healthcare-12-00816-s001.zip › healthcare-2916939-supplementary.pdf]

Health related questionnaire

1. ID:
2. Last Name:
3. First Name:
4. Personal Identification Number (CNP):
5. Age:
6. Phone Number:
7. Interview Location:
8. Place of Residence:
9. Height (cm):
10. Weight (kg):
11. General Practitioner: Yes No
12. Health Insurance: Yes No
13. Last Visit to a Medical Service (approximate date):
14. Emergency: Yes No
15. Employment: Yes No
16. Stable Employment (last 12 months): Yes No
17. Employment Contract: Yes No
18. Occupation (field):
19. Work Abroad: Yes No
20. Education: none primary school lower secondary school upper secondary school
21. Literacy Level (reading/writing): Yes No
22. Smoking: Yes No
23. Alcohol Consumption: Yes No
24. High-Risk Alcohol Consumption (over 5 drinks/week; over 2 units): Yes No
25. Consumption of Sugary Drinks (over 500 ml/day): Yes No
26. History of Pulmonary Pathology: Yes No
27. Pulmonary Pathology:
28. History of Cardiovascular Pathology: Yes No
29. Cardiovascular Pathology:
30. History of Metabolic Pathology: Yes No
31. Diabetes Mellitus: Yes No
32. Metabolic Pathology:
33. History of Digestive Pathology (hepatic or gastrointestinal): Yes No
34. Digestive Pathology:
35. History of Oncological Pathology: Yes No
36. Oncological Pathology:
37. History of Infectious Pathology: Yes No
38. Parasitic Infections: Yes No
39. History of Tuberculosis: Yes No

40. History of COVID-19: Yes No
41. History of Trauma: Yes No
42. Chronic Medication: ! antibiotics
43. Digestive Complaints: Yes No
44. Symptoms: Transit Disorders: Yes No Diarrhea: Yes No Constipation: Yes No Rectal Bleeding: Yes No Abdominal Pain: Yes No Anal Itching: Yes No Nasal Itching: Yes No Heartburn: Yes No Weight Loss: Yes No
45. Stool Sample: Yes No
46. Stool Sample Result:
47. Blood Sugar: random fasting refusal
48. Blood Sugar: mg/dl
49. Hyperglycemia at Diagnosis: Yes No
50. Blood Pressure: mmHg
51. Hypertension at Diagnosis: Yes No
52. Hepatic Steatosis: Yes No
53. Other Pathologies at Initial Diagnosis with Evaluation:
